# Psychological Effects of Whole-body Electromyostimulation Training: a Controlled Pilot Study in Healthy Volunteers

**DOI:** 10.1186/s40798-021-00325-7

**Published:** 2021-06-09

**Authors:** Christian Keicher, Lena Pyrkosch, Bernd Wolfarth, Andreas Ströhle

**Affiliations:** 1 Charité – Universitätsmedizin Berlin, corporate member of Freie Universität Berlin and Humboldt-Universität zu Berlin, Klinik für Psychiatrie und Psychotherapie, Charitéplatz 1, Berlin, 10117 Germany; 2 Charité Research Organisation GmbH, Charitéplatz 1, Berlin, 10117 Germany; 3 Charité – Universitätsmedizin Berlin, corporate member of Freie Universität Berlin and Humboldt-Universität zu Berlin, Abteilung Sportmedizin, Philippstraße 13, 10115 Berlin, Germany

## Abstract

**Background:**

Whole-body electromyostimulation (WB-EMS) training is used in popular and health sports to improve muscular performance. Little is known about the possible psychological effects of WB-EMS training. The aim of the study is therefore to investigate the possible psychological effects of WB-EMS training on subjective well-being, relaxation, mood, and perceived stress.

**Materials and Methods:**

Twenty-five healthy subjects underwent conventional WB-EMS training and Sham training (without the application of electrical stimulation) as part of a randomized, controlled pilot study in a crossover design. Subjective well-being and subjective relaxation were assessed using visual analog scales, the current state of mood was assessed with Multidimensional Mood State Questionnaires (MDBF), and the current level of stress was assessed with Recovery–Stress Questionnaires/Erholungs-Belastungs-Fragebögen (RESTQEBF) before and after training.

**Results:**

WB-EMS training has a statistically significant positive effect on subjective well-being and subjective relaxation, as well as on the awake subscale of the MDBF. No significant main effect of sequence and no interaction effects were found. Also, compared to a Sham training session, a single WB-EMS training session had no significant effect on mood, nervousness, or the current level of stress.

**Conclusion:**

Besides physiological effects, WB-EMS might also have a strong psychological impact. WB-EMS could be beneficial for people who, due to their limitations, have problems training on a regular basis and with adequate training intensity.

**Trial Registration:**

German Clinical Trials Register, DRKS00012583, 22 June 2017.

## Key Points

- Studies on possible psychological effects of WB-EMS training are very few.
- Besides physiological effects, WB-EMS might also have a positive influence on psychological parameters such as subjective well-being and subjective relaxation.
- WB-EMS might be particularly useful for patients who, due to their limitations, have problems training on a regular basis and with adequate training intensity.

## Introduction

Whole-body electromyostimulation (WB-EMS) training has become increasingly popular among the general population in recent years. Despite its rapid development, however, WB-EMS training is more than just a trend sport. The beginning of today’s EMS training goes back to the 1960s [1]. The first scientific description of EMS in competitive sports was published in 1971 by Kots and Chwilon [1]. Published scientific studies with untrained and trained subjects showed a strong effect of WB-EMS training on the parameters of muscular performance [2–12]. However, scientific studies on the psychological effects of WB-EMS training have been largely missing so far. In a recently published study by Jee [13], the efficacy and safety of WB-EMS training on the human body were investigated in 64 healthy volunteers: 31 in a WB-EMS training group and 33 in a control group. In addition to cardiopulmonary parameters (heart rate, systolic blood pressure, diastolic blood pressure, oxygen uptake), psychophysiological variables (pain, anxiety, fatigue, and insomnia) were also assessed. The study concludes that the use of WB-EMS training does not negatively affect cardiopulmonary or psychophysiological factors. Six weeks of WB-EMS training can improve the systolic blood pressure and oxygen uptake in exercise ergometry, as well as psychophysiological factors.

In a recently published study by Eddolls et al. [14], the relationship between physical activity, fitness, and body mass index in relation to mental well-being and quality of life was examined in more detail in a total of 576 adolescents. The study concludes that increased physical activity, cardiorespiratory fitness, and body mass index are directly and indirectly associated with physical well-being and quality of life. An improvement in cardiorespiratory fitness and normalization of body mass index through increased physical activity could have a beneficial effect on the psychological well-being and quality of life of adolescents.

The aim of our present study is to describe the psychological effects of WB-EMS training in more detail using a controlled pilot study with healthy volunteers. The focus of this work is on the areas of subjective well-being, subjective relaxation, current state of mood, and current level of stress. In contrast to the study by Jee [13], we examined psychophysiological factors (not psychopathological symptoms such as soreness, anxiety, fatigability, and sleeplessness) in order to assess healthy psychological status.

## Materials and Methods

### Study Design

The present study is a randomized controlled trial with a standardized experimental design (crossover design).

The study participants were randomized to one of two possible training sequences (EMS or Sham training in the first or second week, respectively). Random numbers from 0 to 9 were generated using a random number generator (https://www.random.org): numbers 0, 1, 2, 3, and 4 were assigned to the EMS–Sham training group (EMS in the first week and Sham in the second week) and numbers 5, 6, 7, 8, and 9 to the Sham–EMS training group (Sham in the first week and EMS in the second week). We did not apply any matching criteria for randomization.

### Recruitment

To initiate and conduct the study, there was a positive ethics vote (EA2/082/17) by the Ethics Committee of Charité, Universitätsmedizin Berlin. The study was performed in accordance with the standards of ethics outlined in the Declaration of Helsinki. The study participants were recruited from two WB-EMS fitness studios in Berlin.

### Inclusion and Exclusion Criteria

Healthy women and men between the ages of 18 and 65 years (both age limits included) could participate in the study. In order to avoid variations in the study results due to the first or multiple applications of WB-EMS training, only persons who regularly perform WB-EMS training once or twice a week were considered. An EMS experience for at least 3 months was needed to participate in the study. Subjects also needed to be able to communicate with the trainer or study staff in German and to fill out the questionnaires and scales. Subjects were asked not to make any lifestyle changes during the running study, such as changes in physical activity or nutrition. Another prerequisite for study participation was good health, characterized as absence of the diseases listed in the following exclusion criteria. The exclusion criteria take into account the recommended contraindications [15] of WB-EMS training and were checked by Anamnese interview before including the subject in the study. Subjects with electronic pacemakers or general electronic implants, acute inflammations, feverish diseases, cancer, untreated arterial hypertension, thrombosis, bleeding or bleeding tendency, stroke, neurological disorders such as epilepsy, muscular dystrophy, paresis, severe liver/kidney disease, and pregnant women were excluded.

### Experimental Procedure

First of all, each participant was informed about the course of the experiment, the duration of the study, the study objective, inclusion/exclusion criteria, and possible risks of participating in the study based on written participant information. In addition, individual questions could be asked in a personal information discussion with a study staff member. After clarification of all open questions, the study participant and the study staff signed the consent form. A signed copy of the written informed consent was given to the subject.

Subsequently, the inclusion and exclusion criteria were examined by the study staff member in an interview with the study participant. If the subject was eligible to enter the study, randomization took place in one of the two training groups.

Apart from the different training conditions, the processes in the gym and also the sequence of the individual scales or questionnaires were unchanged in both weeks. Training in the second week was carried out at the same time of day and with the same personal trainer as in the first week as far as this was possible for the study participant and the trainer. In all cases, there were exactly 7 days between the two training sessions.

### EMS Training

Training equipment from Miha Bodytec (Miha Bodytec GmbH, Siemensstrasse 1, 86368 Gersthofen, Germany; email: info@miha-bodytec.de) was used in our study. Electrostimulation was carried out via electrodes on the upper arms and upper legs. In addition, subjects wore a vest and a belt around the buttock with electrodes. All large muscle groups were involved and the WB-EMS training was under the guidance and supervision of a qualified and experienced personal trainer. The EMS training intensity (in mA) was regulated by the personal trainer, who knew the training condition (EMS versus Sham training) and therefore was not blinded. All personal trainers had a certified training education and several years of experience in WB-EMS training. Safety and motivation were checked by a study staff member during every training session. The trainer trained a maximum of two study participants simultaneously. In addition to the low frequency WB-EMS, isometric and simple dynamic exercises (e.g., squats, ski jumping exercise, etc.) were completed with the following device settings:

- Training duration: 20 min
- Pulse frequency: 85 Hz
- Pulse width: 350 μs
- Pulse duration: 4 s
- Pulse break: 4 s
- Rectangular pulse rise, bipolar

### Sham Training

Study participants wore EMS functional clothing and the WBS-EMS electrodes were connected to the EMS device but, in contrast to the EMS training condition, no impulses were administered. The same isometric hold exercises and dynamic exercises were performed as with the EMS training condition. Using the light-emitting diodes on the EMS device as a feedback instrument, the same interval times (4 s active; voluntary muscle contraction; 4 s break) were performed as in the WB-EMS training.

### Visual Analog Scales (VAS)

The study participants were asked to rate their current subjective well-being or current subjective relaxation. The VAS were initiated with the question “How do you rate your current well-being on a scale of 0 to 100%?” or “What do you think about your current relaxation on a scale of 0 to 100%?” On a straight 100-mm line, with the left end of the line labeled 0% and the right end labeled 100%, subjects with a vertical line assessed their well-being or relaxation on a continuum between 0 and 100.

The position marked by the person (distance from zero, in mm) was used as an indicator of the characteristic expression (subjective well-being, subjective relaxation). The VAS for the current subjective well-being or current subjective relaxation was assessed directly before training and afterwards in the fitness studio.

VAS have a long history as a measuring instrument for researching subjectively assessed characteristics (e.g., mood, satisfaction, pain) in many areas of research; the first VAS were published in the 1920s [16]. The strengths and limitations with regard to the reliability of VAS are clearly addressed in a critical review [16].

### Multidimensional Mood State Questionnaires (MDBF)

The Multidimensional Mood State Questionnaires by Steyer, Schwenkmezger, Notz, and Eid [17] are a measuring instrument for recording three bipolar dimensions of the current mood state: good–bad, awake–tired, and calm–nervous. The test authors prefer the term “mood state questionnaires” to include all three dimensions mentioned above and not just the first one (good–bad). For this study, the trial version (short form B) was selected with a total of 12 items. The MDBF were measured directly before training and afterwards in the fitness studio. The items consist of simple adjectives such as “tired” and “well”. The individual adjectives are assessed on a five-level Likert scale with the endpoints 1 (“not at all”) and 5 (“very much”). The summarized values of the individual scales are calculated from four evaluated adjectives each, whereby a maximum score of 20 can result for each of the three mood scales (good–bad, awake–tired, calm–nervous). Cronbach’s alpha (a measure of internal consistency) for the individual scales ranges between .73 and .89 in this short version.

In a review of the test, Heinrichs and Nater [18] come to the conclusion that, despite its shortness, the method has surprisingly good test quality criteria and is a carefully designed and validated instrument for recording current mood states.

### Recovery–Stress Questionnaires/Erholungs-Belastungs-Fragebögen (RESTQEBF)

The RESTQEBF by Kallus and Kellman [19] capture the person’s stress and recovery activities. For this study, the test version for athletes with a total of 52 items was selected. The procedure asks: “What happened in the last 3 days/nights?.” It reproduces a differentiated picture of the current degree of stress with respect to 12 non-specific (general stress, emotional stress, social stress, conflicts/pressure, fatigue, lack of energy, physical complaints, success, social recovery, physical recovery, general well-being, sleep quality) and 7 sport-specific dimensions (disturbed breaks, emotional exhaustion, injury, being in shape, personal accomplishment, self-efficacy, self-regulation). The RESTQEBF were measured before training in the fitness studio and approximately 72 h later at home. The current level of exposure is estimated by quantitative survey of the frequency of recovery and stress activities over the past 3 days/nights. Due to the behavioral picture of the extent of stress or degree of recovery over the time interval of the last 3 days/nights, the values obtained are largely independent of very short-term and minor changes of state [20].

Cronbach’s alpha for the 19 subtests ranges between .67 and .89 and the retest reliability is above *r* = .79; therefore, it can be assumed that there are well reproducible interindividual differences in the recovery–stress balance.

### Statistical Analyses

Statistical analyses (descriptive statistics, analysis of variance) were carried out using IBM SPSS Statistics Version 23. Unless otherwise stated, a significance level of *p* = .05 was used. All data are reported as the mean (*M*) and standard deviation (*SD*). Both the *F* test (degrees of freedom, *df*) and effect size *f* were calculated. Sphericity was given and no post hoc analyses or baseline adjustments were performed. Outcome criteria were the mean changes (post-minus pre-training values) in psychological variables (subjective well-being, subjective relaxation, MDBF subscales, RESTQEBF). Repeated-measures analyses of variance were performed with treatment (EMS vs. Sham training) as the within-subjects factor and sequence (EMS–Sham vs. Sham–EMS training) as the between-subjects factor. The Kolmogorov-Smirnov test was used to determine the normality of distribution for the examined variables.

## Results

**Fig. 1:**
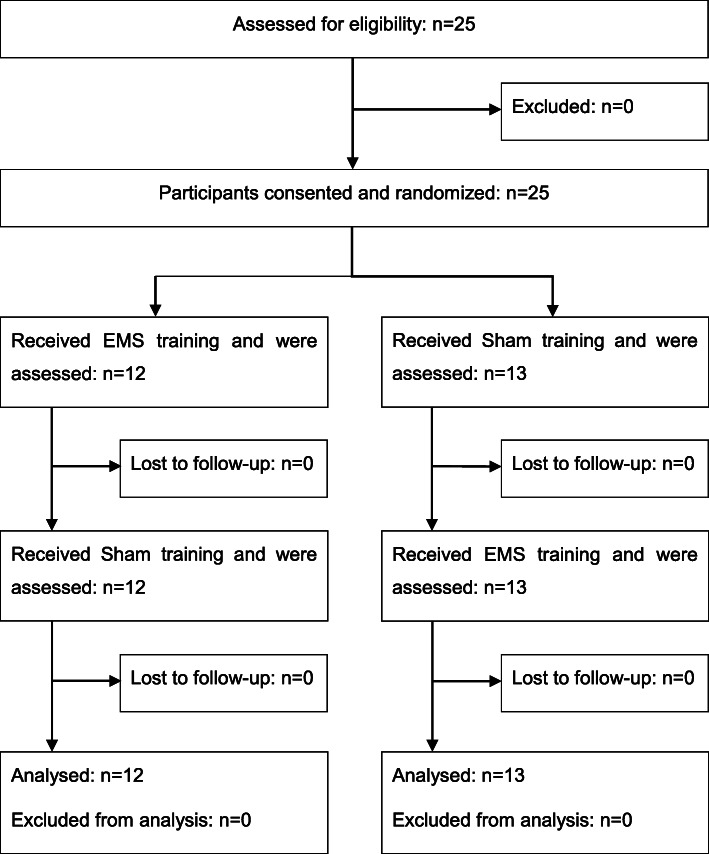
Flow diagram. Abbreviations: *EMS* electromyostimulation, *Sham *control training (without the application of electrical impulses)

Twenty-five healthy subjects (7 women, 18 men) were included in the study from May 2017 through to January 2018. There were no dropouts (Fig. 1). In all our cases, there were exactly 7 days between the two training sessions. No clinically relevant adverse events related to study treatment or study procedure were reported. The average age was 48 (*SD* = 6.4) years. The descriptive statistics and baseline values are depicted in Table 1. There were no statistically significant group differences (except for a discrete difference in the baseline value for physical complaints).

**Table 1:** Descriptive statistics and baseline values

	EMS–Sham (***N*** = 12) ***M(SD)/N***	Sham–EMS (***N*** = 13) ***M(SD)/N***	***t/chi***^***2***^	***df***	***p***
**Age**	47.58(9.88)	48.85(6.59)	–0.37	18.97	.71
**Gender**					
Male	10	8	1.47^a^	1	.38
Female	2	5			
**Subjective well-being**	68.42 (25.21)	65.62 (26.60)	0.27	23	.79
**Subjective relaxation**	64.17 (25.66)	65.77 (25.24)	–0.16	23	.88
**MDBF**					
Awake–tired	12.17 (3.30)	12.46 (3.62)	–0.21	23	.83
Good–bad	16.00 (2.76)	16.15 (1.77)	–0.17	23	.87
Calm–nervous	14.25 (2.80)	15.62 (3.15)	–1.14	23	.27
**RESTQEBF**					
General stress	1.75 (1.22)	1.77 (1.92)	–0.03	23	.98
Emotional stress	2.08 (1.00)	2.92 (1.92)	–1.33	16.48	.20
Social stress	2.08 (1.08)	3.08 (1.85)	–1.62	23	.12
Conflicts/pressure	2.58 (2.43)	3.54 (2.57)	–0.95	23	.35
Fatigue	3.08 (1.88)	4.17 (2.89)	–1.09	18.91	.29
Lack of energy	2.75 (1.55)	2.54 (2.18)	0.28	23	.78
Physical complaints	1.33 (1.30)	2.85 (1.99)	–2.23	23	.04
Success	5.17 (2.86)	5.46 (2.50)	–0.28	23	.79
Social recovery	6.67 (2.31)	7.69 (1.80)	–1.25	23	.23
Physical recovery	7.17 (1.34)	7.00 (1.78)	0.26	23	.80
General well-being	8.17 (1.59)	8.15 (1.57)	0.02	23	.98
Sleep quality	7.58 (2.50)	6.85 (3.29)	0.63	23	.54
Disturbed breaks	3.83 (3.49)	3.38 (2.82)	0.36	23	.73
Emotional exhaustion	1.75 (3.70)	0.92 (1.44)	0.73	22	.48
Injury	4.08 (3.42)	4.50 (4.83)	–0.24	22	.81
Being in shape	13.25 (2.93)	13.38 (4.43)	–0.09	23	.93
Personal accomplishment	10.58 (6.30)	12.17 (5.81)	–0.64	22	.53
Self-efficacy	13.33 (4.91)	13.00 (5.76)	0.15	21	.88
Self-regulation	7.67 (6.53)	8.83 (7.36)	–0.41	22	.69

*Abbreviations*: *EMS* electromyostimulation, *Sham* control training (without the application of electrical impulses), *MDBF* Multidimensional Mood State Questionnaires, *RESTQEBF* Recovery–Stress Questionnaires/Erholungs-Belastungs-Fragebögen, *N* number of subjects, *M(SD)* mean (standard deviation), *t/chi*^*2*^ t/chi^2^ test, *df* degrees of freedom, *p* p value

^a^Two cells had an expected count of less than 5, so Fisher’s exact test was used

There was a statistically significant increase in subjective well-being, relaxation, and MDBF awake–tired mood in the EMS training condition compared to the Sham training condition (*F* > 5.10, *df* = 1, 23, *p* < 0.03). Further details are displayed in Table 2. There were no significant main effects of sequence and no significant interaction of sequence by treatment. Furthermore, there were no significant main or interaction effects for the other MDBF subscales (good–bad, calm–nervous) or RESTQEBF.

**Table 2:** Repeated measures analyses of variance for different outcome criteria

Clinical scale	Effect	Group	***N***	Pre ***M(SD)***	Post ***M(SD)***	∆ ***M(SD)***^**a**^	***F(df)***	***p***	***f***
**Subjective well-being**	Treatment	EMS	25	64.12 (22.43)	84.28 (13.29)	20.16 (16.75)	7.85 (1.23)	.01*	0.58**
		SHAM	25	66.68 (28.21)	74.80 (23.12)	8.12 (18.52)			
	Sequence	EMS–SHAM	12				1.86 (1.23)	.19	0.28*
		SHAM–EMS	13						
	Treatment * sequence						0.24 (1.23)	.63	0.10
**Subjective relaxation**	Treatment	EMS	25	64.12 (20.95)	79.04 (15.60)	14.92 (19.63)	5.10 (1.23)	.03*	0.47**
		SHAM	25	64.56 (27.´´35)	70.60 (24.97)	6.04 (12.62)			
	Sequence	EMS–SHAM	12				0.22 (1.23)	.65	0.10
		SHAM–EMS	13						
	Treatment * sequence						1.75 (1.23)	.20	0.28*
**MDBF** **awake**–**tired**	Treatment	EMS	25	12.60 (3.98)	17.16 (1.80)	4.56 (4.02)	25.75 (1.23)	<.001***	1.06**
		SHAM	25	14.80 (3.00)	14.88 (2.11)	0.08 (2.90)			
	Sequence	EMS–SHAM	12				1.84 (1.23)	.19	0.28*
		SHAM–EMS	13						
	Treatment * sequence						0.50 (1.23)	.49	0.15
**MDBF** **good**–**bad**	Treatment	EMS	25	16.04 (2.44)	17.60 (1.78)	1.56 (2.99)	0.40 (1.23)	.53	0.13
		SHAM	25	15.76 (2.79)	16.96 (2.19)	1.20 (1.91)			
	Sequence	EMS–SHAM	12				1.07 (1.23)	.31	0.22
		SHAM–EMS	13						
	Treatment * sequence						2.60 (1.23)	.12	0.34*
**MDBF** **calm**–**nervous**	Treatment	EMS	25	14.84 (2.62)	16.80 (2.21)	1.96 (2.96)	2.45 (1.23)	.13	0.33*
		SHAM	25	14.72 (3.34)	15.80 (2.99)	1.08 (1.75)			
	Sequence	EMS–SHAM	12				0.74 (1.23)	.40	0.18
		SHAM–EMS	13						
	Treatment * sequence						0.81 (1.23)	.38	0.19
**RESTQEBF**	Treatment	EMS	25	5.88 (1.32)	5.89 (1.42)	0.01 (1.08)	0.19 (1.23)	.66	0.09
		SHAM	25	5.84 (1.54)	5.78 (1.40)	-0.06 (0.73)			
	Sequence	EMS–SHAM	12				0.46 (1.23)	.50	0.14
		SHAM–EMS	13						
	Treatment * sequence						3.20 (1.23)	.08	0.37*

*Abbreviations*: *EMS* electromyostimulation, *Sham* control training (without the application of electrical impulses), *MDBF* Multidimensional Mood State Questionnaires, *RESTQEBF* Recovery–Stress Questionnaires/Erholungs-Belastungs-Fragebögen, *N* number of subjects*, Pre M(SD)* pre-treatment mean (standard deviation), *Post M(SD)* post-treatment mean (standard deviation), *F(df) F* test (degrees of freedom), *p* p value, *f* effect size

^a^∆ = difference measured between pre- and post-treatment

*p* < .05: significant*; *p* < .01: very significant**; *p* < .001: highly significant***

*f* = .10: small effect; *f* = .25: medium effect*; *f* = .40: large effect**

**Fig. 2:**
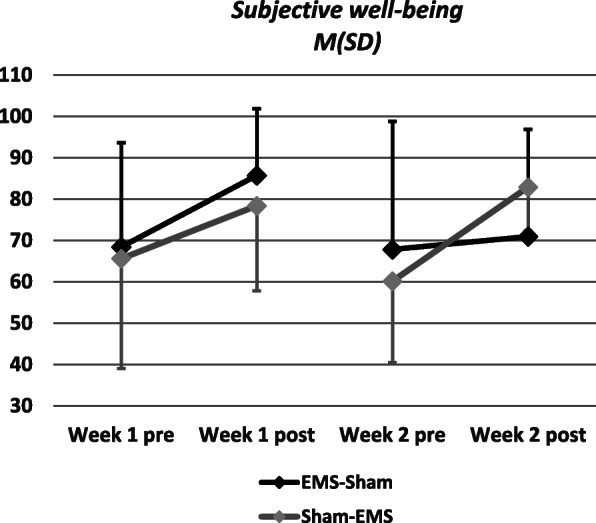
Change in subjective well-being over training period. Abbreviations: EMS, electromyostimulation, Sham control training (without the application of electrical impulses), M(SD) mean (standard deviation)

Figure 2 visualizes subjective well-being (*M* ± *SD*) measured with the VAS at four timepoints (week 1 pre, week 1 post, week 2 pre, week 2 post). Although there was no statistically significant interaction effect, Fig. 2 shows that in the first week subjective well-being increases virtually irrespective of training mode (with or without electrical stimulation). However, in the second week subjective well-being was virtually only affected in the active group, indicating that subjects in the Sham group realized a difference to the first week when they had the active intervention. As we did not find a statistically significant interaction effect, we cannot recommend the regimen of exercising without stimulation first and then adding stimulation later to achieve the biggest effects. Furthermore, all of our study participants already had experience with WB-EMS training.

## Discussion

The main result of our study is that a single WB-EMS training session has significant psychological effects: compared to Sham training, subjective well-being, subjective relaxation, and awakeness increased in a group of healthy subjects. No direct effects on mood (calm–nervous) or the current level of stress could be found.

This study is the first to examine the psychological effects of a single WB-EMS training session. The psychological effects of a 6-week WB-EMS session have been published recently by Jee [13], where a significant improvement in psychophysiological factors (soreness, anxiety, fatigability, and sleeplessness) is described. However, the study raises some methodological ambiguities. On the one hand, the study included students aged 20–25 years and it is questionable whether students represent a representative sample for the collection of the examined psychophysiological parameters. In addition, it is not clear how a statistically significant improvement of psychophysiological factors can occur because the students are healthy study participants and the psychophysiological factors examined in the study are complaints and/or symptoms of diseases (soreness, anxiety, fatigability, and sleeplessness). In order to be able to measure an improvement, there should initially be abnormalities in the examined parameters. In addition to the group of WB-EMS exercisers, there is a control group that also trained for 6 weeks in the study. In the control group, the same exercises were performed in EMS suits but without the application of impulses. It is a pity that only the intervention group with WB-EMS training was examined for psychophysiological parameters. There are no comparative data on possible psychophysiological changes in the control group (which were also physically active).

A publication by Harvey, Bradley, and Aronica from 1992 [21] examined the psychological effects of functional EMS in patients with spinal cord injury; the functional EMS served to train the mobility and function of muscles atrophied as a result of spinal cord injury. In addition to the improvement of physiological parameters, a possible psychological benefit in spinal cord patients was discussed in the study [21].

Our results also suggest that besides the physiological effects, WB-EMS might serve as an intervention with strong psychological effects. On the basis of our study results, we can give only an outlook on the short-term effects of WB-EMS training. We believe that WB-EMS training will also have positive long-term effects on psychophysiological factors but this point would need to be tested in further studies.

Compared to standard resistance training, WB-EMS has the advantage of being more time-efficient. Kemmler et al. have shown that WB-EMS training is an effective, time-saving form of strength training that, when performed regularly, can achieve comparable results to conventional strength training [2–7]. Furthermore, the effort for the subjects is reduced and this might be the reason for the common popularity of this form of training in healthy subjects, leading to an explosion of studios in some countries such as Germany.

Notwithstanding the positive effects of WB-EMS training, however, possible risks and dangers of WB-EMS training should also be considered. In a recently published review by Stöllberger and Finsterer [22], data on indications and side effects of WB-EMS training were systematically evaluated. The study reports excessive post-exercise creatine kinase elevations as a serious side effect of WB-EMS training. Especially after the first application, there is a risk of rhabdomyolysis with consecutive acute kidney damage. Highly intensive WB-EMS applications should therefore be avoided at the beginning of the training process. In our study, we wanted to examine the effects of a moderate to challenging WB-EMS training session. Because of the possible side effect of rhabdomyolysis after initial intensities that are too high, we chose non-beginners for WB-EMS training in our study. In the medical field, the question arises whether WB-EMS might be beneficial for patients as well. For instance, depressed or elderly people often have problems playing sport due to physical or mental limitations. There is an increasing amount of evidence that sport, physical activity, and structured exercise programs improve the physical and psychological well-being of people with psychosocial disabilities [23]. Our data provide the first evidence for positive psychological effects of WB-EMS training in healthy volunteers, with the question arising as to whether WB-EMS might be used in patients to induce not only physiological effects but also positive psychological effects on the symptoms of the underlying disease. Therefore, further studies in patients with mental disorders are needed.

The major limitations of our study are the small sample size, the non-clinical sample (which makes it difficult to find positive psychological effects), the potential for selection bias, and the difficulties in blinding of the Sham intervention. Originally, it was planned to perform placebo training as a control. However, study participants should not be informed that they are training without a current impulse; therefore, the phrase “current pulse below the perceptible stimulus limit” was chosen on the subject information form. This planned approach was also approved by the Ethics Committee of Charité, with a positive ethics vote. In practice, however, the personal trainers refused to tell their clients (and, in part, their long-term customers) that an imperceptible current pulse was administered during the Sham control condition. In addition, there is potential for selection bias in our study. The study participants had to have at least 3 months’ training in the WB-EMS experience. If participants did not enjoy or respond favorably to WB-EMS or they experienced adverse events, they would not continue with WB-EMS training for more than 3 months. The study population therefore might be selectively biased toward reacting favorably to WB-EMS training. If we had WB-EMS naïve subjects, we may have obtained less favorable results. Another weakness of this pilot study is that the given randomization list could not be 100% complied with. There were seven study participants who determined the order of the two training conditions for themselves during the study: they threatened not to participate in the study if their request was not taken into account; therefore, the randomization list was adapted accordingly. As we did not apply any matching criteria for randomization, there was an unbalanced gender ratio between both training groups. However, there were no statistically significant group differences (except for a discrete difference in the baseline value for physical complaints). Taken together, our results suggest that besides the physiological effects, WB-EMS might also have a strong positive impact on the mental state. The improvement of physical factors such as strength by WB-EMS could be accompanied by psychological changes in a positive sense.

## Conclusions

Although WB-EMS training is used in popular and health sports to improve muscular performance, little is known about the possible psychological effects of WB-EMS training. Therefore, the aim of this study was to investigate the possible psychological effects of WB-EMS training on subjective well-being, relaxation, mood, and perceived stress. Our results indicate that WB-EMS has a statistically significant effect on subjective well-being and subjective relaxation. Furthermore, our pilot study provides the first evidence that, besides the known and published physiological effects, WB-EMS might also have positive psychological effects. To our knowledge, this study is the first to examine the psychological effects of a single WB-EMS training session. Additional studies are undoubtedly needed to gather more data and develop a clearer picture of the trends we have demonstrated in our pilot study. WB-EMS might also be used in patients with a mental disorder who, due to their limitations, have problems training on a regular basis and with adequate training intensity.

## Data Availability

Please contact the corresponding author for data requests.

## Abbreviations

*df*: Degrees of freedom
EMS: Electromyostimulation
*M*: Mean
MDBF: Multidimensional Mood State Questionnaires
*N*: Number of subjects
RESTQEBF: Recovery–Stress Questionnaires/Erholungs-Belastungs-Fragebögen
*SD*: Standard deviation
VAS: Visual analog scales
WB-EMS: Whole-body electromyostimulation
